# Reactivation of occult hepatitis B virus infection in a renal transplant recipient

**DOI:** 10.1186/s12985-022-01946-4

**Published:** 2022-12-15

**Authors:** Lili Jiang, Huiqi Wang, Yaping Huang, Hanying Liang, Xiaodong Wang, Jun Fan

**Affiliations:** State Key Laboratory for Diagnosis and Treatment of Infectious Diseases, Collaborative Innovation Center for Diagnosis and Treatment of Infectious Diseases, The First Affiliated Hospital, Zhejiang University School of Medicine, Hangzhou, Zhejiang China

**Keywords:** HBV reactivation, Occult infection, HBV escape mutant, Immunity, Renal transplantation

## Abstract

We report a case of hepatitis B virus (HBV) reactivation in a renal transplant recipient. Reactivation manifested as an occult infection with detectable HBV-DNA and negativity for hepatitis B surface antigen (HBsAg). The anti-HBs antibody titre was above the protective threshold and continued to rise, to 951.36 mIU/ml, after HBV reactivation. Sequencing revealed multiple vaccine- and diagnostic-escape mutations in the major hydrophilic region of HBsAg. This case demonstrates both reactivation of an HBV escape mutant in a vaccinated patient and host immunity after virus mutation.

**Supplementary Information:**

The online version contains supplementary material available at 10.1186/s12985-022-01946-4.

## Introduction

Renal transplantation (RT) is the first-choice treatment for patients with end-stage renal disease [1]. Following RT, rejection is prevented by drugs or biological agents [2, 3]. In these immunosuppressed patients, hepatitis B virus reactivation (HBVr) is not uncommon, and it may lead to severe acute hepatitis, fulminant liver failure, and death [4–6]. Occult HBV infection (OBI), defined as the detection of HBV-DNA in the liver and/or serum in HBsAg-negative individuals, has attracted considerable attention in recent years [7, 8].

Here, we report a case of HBVr manifesting as OBI in a renal transplant recipient despite a pre-existing protective anti-HBs antibody titre. A rare HBV serological spectrum was determined, with further analysis showing that the activated virus was an escape mutant.

## Case presentation

A 71-year-old male patient previously diagnosed with congenital polycystic kidney disease and hypertensive nephrosclerosis had been vaccinated against HBV. He underwent RT on November 27, 2019. Pre-transplant HBV serological analysis showed that he was positive for anti-HBc and anti-HBs (146.91 mIU/ml). In December 2020, HBV-DNA was detected serum, although HBsAg and HBeAg were negative in serum. Two weeks later, the HBV viral load increased to 74.5 IU/ml, and HBeAg became weakly positive. In February 2021, he received nucleoside antiviral drugs (tenofovir alafenamide, TAF) once his HBV viral load (2440 IU/ml) exceeded 2000 IU/ml, and once he became clearly HBeAg positive. One month after antiviral therapy, serum HBV-DNA became undetectable, and HBeAg showed seroconversion (HBeAg became negative and HBeAb became positive). Antiviral drugs were discontinued after one course of treatment (3 months). At the latest follow-up, HBV-DNA was not detected. The serum level of alanine aminotransferase was always normal. Due to the patient's older age, further HBV virus detection in the patient’s liver tissue and pathological biopsy analysis were not performed.

## Results

### Monitoring of HBV markers

**Fig. 1:**
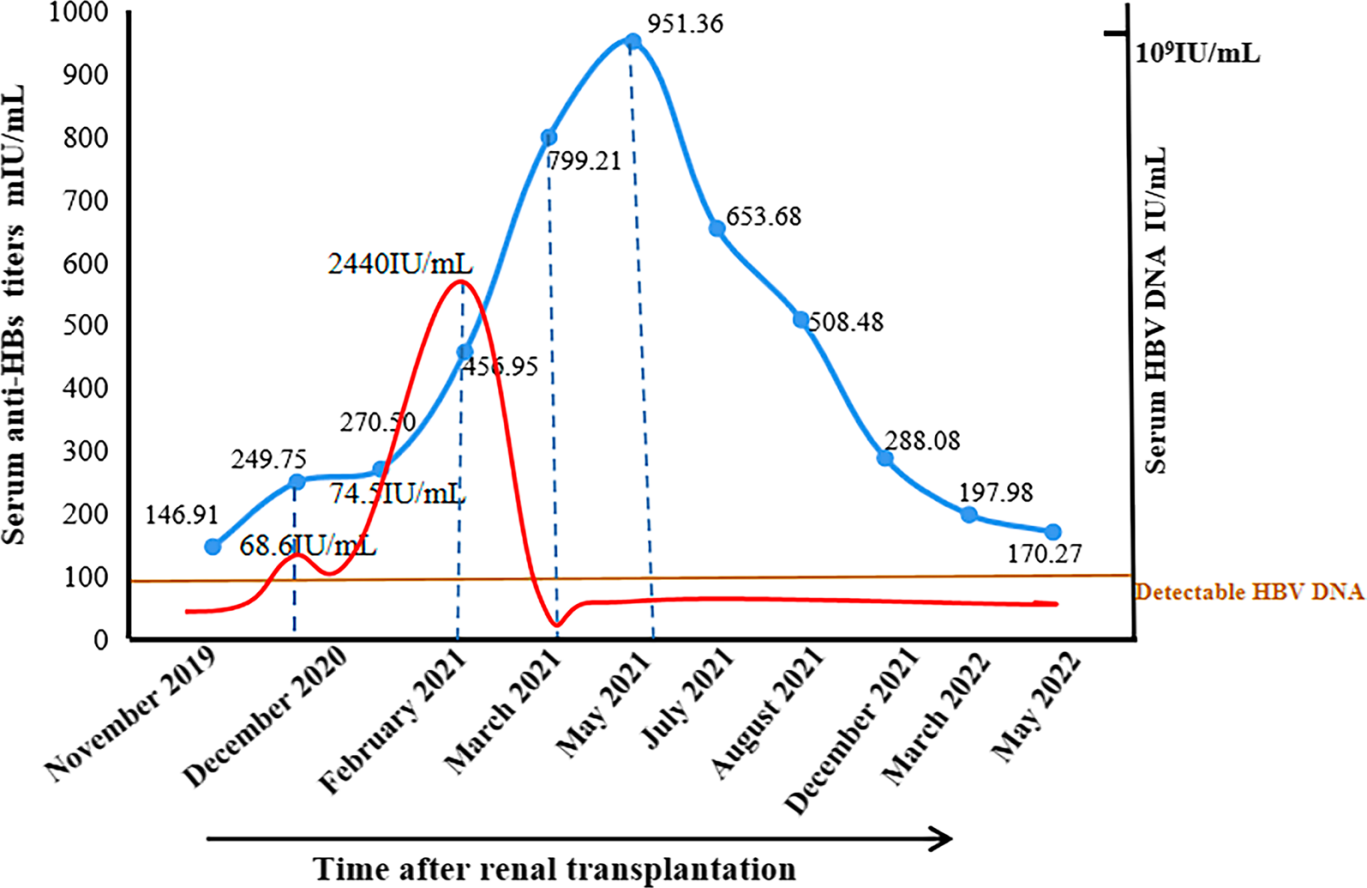
Changes in the serological parameters of HBV infection. Anti-HBs antibody titres in a patient with reactivated HBV increased gradually, following an increase in the HBV viral load, and peaked at 951.36 mIU/ml, at which point HBV-DNA became undetectable. The blue and red lines represent the changes in the anti HBs titre and HBV-DNA level, respectively. The dotted lines indicate the anti-HBs antibody titre and HBV viral load at the following time points: when HBV activation was detected, when antiviral drugs were started, when HBV-DNA was below the detection limit, and when the antibody titre peaked (left to right, respectively)

Detailed data on the HBV infection markers at follow-up in the patient are shown in Table 1. HBV-DNA was not detected, but his anti-HBs antibody titre increased to 951.36 mIU/ml and then decreased (Fig. 1). Given this unusual serological mode of infection, follow-up of the patient will be continued.

**Table 1:** Viral serological markers follow up of hepatitis B reactivation patient

Assay/Month	Pre-trans	Post-trans									
Date	Nov 2019	Dec 2020^1^*	Dec 2020^2^*	Feb 2021	Mar 2021	May 2021	Jul 2021	Aug 2021	Dec 2021	Mar 2022	May 2022
HBV-DNA(IU/ml)	ND*	68.6	74.5	2440	Neg	Neg	Neg	Neg	Neg	Neg	Neg
HBsAg(mIU/ml)	Neg	Neg	Neg	Neg	Neg	Neg	Neg	Neg	Neg	Neg	Neg
HBsAb(mIU/mL)	146.91	249.75	270.5	456.95	799.21	951.36	653.66	508.48	288.08	197.98	170.27
HBeAg(PEIU/ml)	0.16	0.15	0.19	1.02	0.08	0.08	0.08	0.08	0.06	0.06	0.06
HBeAb(s/co)	Neg	Neg	Neg	Neg	Neg	Pos	Pos	Pos	Pos	Neg	Pos
HBcAb(s/co)	Pos	Pos	Pos	Pos	Pos	Pos	Pos	Pos	Pos	Pos	Pos

Dynamic monitoring of serological parameters of HBV infection after renal transplantation. Serological HBV parameters (HBsAg, HBcAb, HBsAb, HBeAg, and HBeAb) were analysed (using kits from Abbott Laboratories) between November 2019 and May 2022. HBV viral load was determined by TaqMan PCR assay (Shanghai Zhijiang Biology) and is expressed in IU/ml (threshold level = 30 IU/ml)

ND* not detected; *Neg* negative in a semi-quantitative assay or HBV viral load below the detection limit

Dec 2020^1^* and Dec 2020^2^*: there were two different testing times in December 2020. In early December 2020, the patient was negative for serum HBsAg but positive for HBV-DNA. Two weeks later, blood was collected again for retesting

### Analysis of the HBV genome

**Fig. 2:**
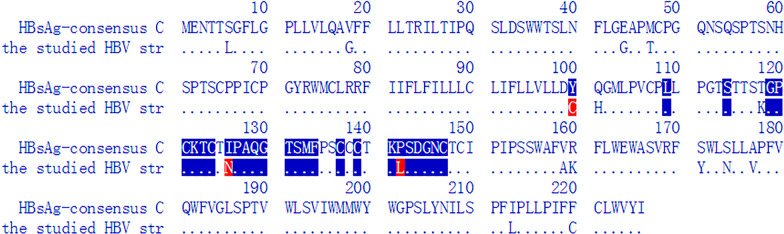
Alignment of the studied HBsAg amino-acid sequence to the closest reference isolate (amino acids 1–226). The studied HBV genome sequence was analysed against online HBV-drug resistance data (http://www.hiv-grade.de/hbv_grade/deployed/grade.pl?program=hbvalg). Above and below are the amino acid sequences of HBsAg for the closest reference isolate and our HBV strain, respectively. The one-letter amino-acid code is shown only for positions different to the consensus sequence. Identical positions are denoted by ".". Positions for which no patient sequence data were available are denoted by "–". Sixteen amino acid substitutions were identified in the HBV surface (S) protein: S6L, V18G, E44G, M47T, Y100C, Q101H, T118K, I126N, P142L, V159A, R160K, S171Y, S174N, A177V, I213L, and F220C. Substitutions of Y100C, I126N, and P142L (red) are HBsAg escape mutations

The complete genome sequence (3215 bp) of the HBV strain evaluated was obtained by nested PCR amplification, sequencing, and splicing. Sequence comparison with an online database showed that the strain belonged to genotype C1 and had 95.62% similarity with the HBV reference genome GQ358158. Further analysis of the virally encoded amino acid sequence yielded the following results: no previously reported resistance mutations; six amino acid substitutions in the reverse transcriptase domain (H126Q, D134E, Q267L, I269L, A317S, and N337H); and 16 amino acid substitutions in HBsAg (amino acids 1–226), comprising 4 (S6L, V18G, E44G, and M47T) in the N-terminal region (amino acids 1–99), 7 (Y100C, Q101H, T118K, I126N, P142L, V159A, and R160K) in the highly conserved major hydrophilic region (amino acids 99–169), and the remainder (S171Y, S174N, A177V, I213L, and F220C) in the C-terminal region (amino acids 170–226) of HBsAg (Fig. 2).

### Determination of the neutralisation efficiency of anti-HBs antibodies

The serum samples of the patient were incubated with normal saline and the recombinant HBsAg (rHBsAg) subtypes adw, adr, and ayw. After neutralisation, anti-HBs antibody titres were 608.77, 1.05, 1.06, and 585.19 mIU/ml, respectively. The reactivities of anti-HBs antibodies with the rHBsAg adw, adr, and ayw subtypes were 99.83%, 99.82%, and 3.87%, respectively.

## Discussion

HBV infection with detectable HBV-DNA but HBsAg negativity was found in a male patient during the first year after RT. The HBV DNA viral load increased from 68.6 to 2440 IU/ml, and HBeAg became obviously positive within 2 months, consistent with reactivation and occult infection [5, 7]. Since vaccination does not lead to the production of anti-HBc antibodies [9], pre-transplant anti-HBc positivity indicates a history of HBV exposure and a long persistence of HBV covalently closed circular DNA in liver cells [10, 11]. As the patient had been taking tacrolimus (FK506) to suppress kidney rejection, immunosuppression-induced reactivation with resolved HBV infection was the most likely diagnosis.

In individuals who have undergone vaccination, anti-HBs antibody concentrations > 100 mIU/ml imply a high level of protection [12]. Previous studies showed that a progressive decline in anti-HBs antibody titres is a predictor of HBVr [13, 14]. However, this was not supported by the HBV serological data in our patient, in whom HBVr occurred in the presence of a high pre-existing anti-HBs antibody titre, which gradually increased to 951.36 mIU/ml after reactivation. Wang et al. [15] reported increases in the post-transplant anti-HBs titres in HBsAg-negative renal transplant recipients who received a kidney from an HBsAg-positive donor. The authors speculated that HBsAg stimulation was similar to HBV “vaccination”. A subsequent neutralisation analysis showed good reactivity of anti-HBs antibodies to the rHBsAg subtypes adw and adr but poor reactivity to the ayw subtype. These findings demonstrate that despite the presence of a high titre and specific anti-HBs antibodies in our patient, HBV reactivation and replication were not inhibited.

To elucidate the underlying mechanism of the OBI, we investigated the genetic features of the HBV strain. An analysis of the complete genome sequence revealed that the virus was genotype C1, serological subtype adw2 [16]. Further analysis of HBsAg amino acids identified 16 substitutions. Substitutions I126N and P142L were located in the “a” determinant region (amino acids 124–147, major target of neutralising antibodies). Both are considered typical mutations associated with the ability of HBsAg to escape detection and vaccine efficacy [17, 18]. Additionally, amino acid substitution at positions Y100C, Q101H, T118K, V159A, and R160K (located in the major hydrophilic region, close to the “a” determinant) may also alter HBsAg antigenicity [19, 20]. HBV strains carrying these HBsAg mutations may escape recognition by diagnostic and protective antibodies, resulting in virus reactivation and diagnostic failure (negative for HBsAg).

However, several questions remain to be considered. First, why were HBV mutations present? Second, why was there no decrease, but rather an increase in anti-HBs antibody levels, after HBVr? The literature suggests that HBV-specific T cells are the main effectors that eliminate infected cells or inhibit viral replication [21, 22], such that treatment with the T-cell inhibitor tacrolimus (FK506) to prevent transplant rejection is associated with a high risk of HBVr [3, 23]. The mutated HBV may reflect selection for immune escape in this vaccinated transplant recipient and thus its efficacy in reactivation [5]. However, wild-type HBV strains that stimulate the proliferation and differentiation of HBV-specific memory B cells, and in turn the production of anti-HBs antibodies, no doubt persisted [16, 24]. Since anti-HBs antibodies are not directed against viral mutants, they have no protective activity.

## Materials and methods

### HBV viral load and monitoring of serological markers

Serological parameters of HBV (HBsAg, HBcAb, HBsAb, HBeAg, and HBeAb) were analysed between November 2019 and May 2022 using kits from Abbott Laboratories (Sligo, Ireland). HBsAg and HBeAg concentrations > 0.05 IU/ml and > 0.18 PEI U/ml, respectively, were considered to indicate HBV positivity. The threshold level of anti-HBs was set at 10 mIU/ml. HBeAb and HBcAb concentration determinations were considered to be semi-quantitative. The HBV viral load was determined by TaqMan PCR assay (Shanghai Zhijiang Biology, Shanghai, China) and is expressed in IU/ml (threshold level = 30 IU/ml). The test reagent batch number was P20220901.

### Analysis of the HBV genome sequence

Serum samples were collected from the patient in February 2021 (HBV-DNA 2440 IU/ml) and stored until HBV genome sequence analysis. Viral DNA was extracted from 200 µl of serum using a DNA extraction kit (Guangzhou Daan Gene, Guangzhou, China). The HBV genome was divided into two fragments (fragments I and II), each of which was amplified by nested PCR as described in the literature [25, 26]. PCR products were identified by agarose gel electrophoresis followed by sequencing (Shanghai Biotechnology). The primers used for amplification and sequencing are listed in Additional files 1 and 2. The full-length gene sequence was obtained by splicing the two segments in Seqman software. The resulting sequence was analysed using the online HBV databases Geno2pheno [hbv] 2.0 (https://hbv.geno2pheno.org/index.php) and HBV-drug resistance interpretation (http://www.hiv-grade.de/hbv_grade/deployed/grade.pl?program=hbvalg).

### Determination of the neutralisation efficiency of anti-HBs antibodies

Serum samples obtained in May 2021 (anti-HBs 653.66 mIU/ml) were stored until used for anti-HBs antibody neutralisation analysis. Serum (200 µl) was incubated with the rHBsAg subtypes adw, adr, and ayw (Prospec, Israel) or, as a control, with normal saline. After 1 h of incubation at 37℃, the supernatant was assayed for anti-HBs antibodies (Abbott Laboratories). The results are expressed as the degree of anti-HBs antibody reactivity, defined as (titre of control − titre of experimental group) ÷ titre of control × 100%.

## Conclusions

This case demonstrates the potential reactivation of an HBV escape mutant, resulting in occult infection. It thus provides insight into the persistence of HBV protective immunity after variant infection. Moreover, it is a reminder of the importance of HBV DNA detection in monitoring immunosuppressive patients with a history of HBV infection. Early recognition of virological replication and initiation of antiviral therapy can prevent further liver damage.

## Supplementary Information

**Additional file 1**. Primers used for HBV genome amplification and sequencing.

**Additional file 2**. Sequence.

## Abbreviations

HBV: Hepatitis B virus
HBsAg: Hepatitis B surface antigen
anti-HBs: Antibodies against HBsAg
anti-HBc: Antibodies against HBcAg
HBeAg: Hepatitis B e antigen
HBeAb: Antibodies against HBeAg
RT: Renal transplantation
HBVr: Hepatitis B reactivation
OBI: Occult HBV infection
rHBsAg: Recombinant HBsAg
