# Supplementary material for: Reactivation of occult hepatitis B virus infection in a renal transplant recipient
Source: Virol J. 2022 Dec 15;19:216. doi: 10.1186/s12985-022-01946-4 (PMC9753329; doi:10.1186/s12985-022-01946-4)
Supplement: Supplementary file 1 — Additional file 1. Primers used for HBV genome amplification and sequencing. [file 12985_2022_1946_MOESM1_ESM.docx]

**Reactivation of occult hepatitis B virus infection in a renal transplant recipient**

Lili Jiang^1*^, Huiqi Wang^1^, Yaping Huang^1^, Hanying Liang^1^, Xiaodong Wang^1^and Jun Fan^1*^

Supplementary materials: Primers used for HBV genome amplification and sequencing.

| **Fragments** |  | **Primer** | **Sequence (5′-3′)** | **Position (nt^a^)** |
| --- | --- | --- | --- | --- |
| Fragment Ⅰ  (nt702-182) | 1st primer set | P3 | 5′-CTCGCTCGCCCAAATTTTTCACCTCTGCCTAATCA-3′ | 1825–1841 |
|  |  | AR1 | 5′ -ACAGTGGGGGAAAGC-3′ | 759-745 |
|  | 2nd primer set | P3 | 5′-CTCGCTCGCCCAAATTTTTCACCTCTGCCTAATCA-3′ | 1825-1841 |
|  |  | AR2 | 5′ -AGAAACGGRCTGAGGC-3′ | 702-687 |
| Fragment II  (nt1823-503) | 1st primer set | AF1 | 5′ -GTCTGCGGCGTTTTATC-3′ | 419-435 |
|  |  | P4 | 5′ -CTGGTTCGGCCCAAAAAGTTGCATGGTGCTGG-3′ | 1823-1806 |
|  | 2nd primer set | AF2 | 5′ -GTCTGCGGCGTTTTATC-3′ | 503-519 |
|  |  | P4 | 5′ -CTGGTTCGGCCCAAAAAGTTGCATGGTGCTGG-3′ | 1823-1806 |
| Fragment Ⅰ  sequencing |  | AR2 | 5′ -AGAAACGGRCTGAGGC-3′ |  |
|  |  | 5P | GAGTAGGCTGTCTTCCTGACTG |  |
|  |  | 6P | CAGCCTTCCACAGAGTATGTAA |  |
| Fragment II  sequencing |  | AF2 | 5′ -GTCTGCGGCGTTTTATC-3′ |  |
|  |  | 3p | AATCCTGAGTGGCAAACTCC |  |

**Note:** Primers used for amplification and sequencing. The HBV genome was divided into two fragments (fragments I and II), each of which was amplified by nested PCR. PCR products were identified by agarose gel electrophoresis followed by sequencing (Shanghai Biotechnology).

**References**

1. Yuan, Q.; Ou Sh Fau - Chen, C.-R.; Chen Cr Fau - Ge, S.-X.; Ge Sx Fau - Pei, B.; Pei B Fau - Chen, Q.-R.; Chen Qr Fau - Yan, Q.; Yan Q Fau - Lin, Y.-C.; Lin Yc Fau - Ni, H.-Y.; Ni Hy Fau - Huang, C.-H.; Huang Ch Fau - Yeo, A.E.T.; et al. Molecular characteristics of occult hepatitis B virus from blood donors in southeast China.

2. Chen, Y.; Qian F Fau - Yuan, Q.; Yuan Q Fau - Li, X.; Li X Fau - Wu, W.; Wu W Fau - Guo, X.; Guo X Fau - Li, L.; Li, L. Mutations in hepatitis B virus DNA from patients with coexisting HBsAg and anti-HBs.
