# Supplementary material for: Reactivation of occult hepatitis B virus infection in a renal transplant recipient
Source: Virol J. 2022 Dec 15;19:216. doi: 10.1186/s12985-022-01946-4 (PMC9753329; doi:10.1186/s12985-022-01946-4)
Supplement: Supplementary file 2 — Additional file 2. Sequence. [file 12985_2022_1946_MOESM2_ESM.docx]

**Reactivation of occult hepatitis B virus infection in a renal transplant recipient**

Lili Jiang^1*^, Huiqi Wang^1^, Yaping Huang^1^, Hanying Liang^1^, Xiaodong Wang^1^and Jun Fan^1*^

Supplementary materials 2: HBV genome sequence.

The HBV genome was divided into two fragments (fragments I and II), each of which was amplified by nested PCR as described in the literature(Chen et al.;Yuan et al.). PCR products were identified by agarose gel electrophoresis followed by sequencing (Shanghai Biotechnology). The primers used for amplification and sequencing are listed in supplementary materials 1. We obtained the 3215 bp whole genome sequence of this HBV strain, see blow:

ATCTCATGTTCATGTCCTACTGTTCAAGCCTCCAAGCTGTGCCTTGGGTGGCTTTGGGGCATGGACATTGACCCGTATAAAGAATTTGGAGCTTCTGTGGAGTTACTCTCTTTTTTGCCTTCTGACTTCTTTCCTTCTATTCGAGATCTCCTCGACACCGCCTCTGCTCTGTATCGGGAGGCCTTAGAGTCTCCGGAACATTGTTCACCTCACCATACAGCACTCAGGCAAGCTATTCTGTGTTGGGGTGAGTTGATGAATCTGGCCACCTGGGTGGGAAGTAATTTGGAAGACCCAGCATCCAGGGAATTAGTAGTCAGCTATGTCAATGTTAATATGGGCCTGAAAATCAGACAACTGCTGTGGTTTCACATTTCCTGTCTTACTTTTGGAAGAGAAACTGTTCTTGAGTATTTGGTGTCTTTTGGAGTGTGGATTCGCACTCCTCCTGCTTACAGACCACCAAATGCCCCTATCTTATCAACACTTCCGGAAACTACTGTTATTAGACGACGAGGCAGGTCCCCTAGAAGAAGAACTCCCTCGCCTCGCAGACGAAGATCTCAATCGCCGCGTCGCAGAAGATCTCAATCTCGGGAATCTCAATGTTAGTATCCCTTGGACTCATAAGGTGGGAAACTTTACTGGGCTTTATTCTTCTACTGTACCTGTTTTTAATCCTGAGTGGCAAACTCCCGCCTTTCCTCATATTCATTTACAGGAGGACATTATTAATAGATGTCAACAATATGTGGGCCCTCTTACAGTTAATGAAAAAAGGAGATTAAAATTAATTATGCCTGCTAGGTTCTATCCTAACCTTACCAAATATTTGCCCTTAGACAAAGGCATTAAACCATATTATCCTGAACATGCAGTTAATCATTACTTCAAGACTAGGCATTATTTACATACTCTGTGGAAGGCTGGCATTCTATATAAGAGAGAAACTACCCGCAGCGCCTCATTTTGTGGGTCACCATATTCTTGGGAACAAGAGCTACAGCATGGGAGGTTGGTCTTCCAAACCTCGACAAGGCATGGGGACAAATCTTTCTGTTCCCAATCCTCTGGGATTCTTTCCCGATCACCAGTTGGACCCTGCGTTCGGAGCCAATTCAAACAATCCAGATTGGGACTTCAACCCCAACAAGGATCATTGGCCAGAGGCAAATCAGGTAGGAGCGGGAGCATTCGGGCCAGGGTTCACCCCACCACACGGCGGTCTTTTGGGGTGGAGCCCTCAGGCTCAGGGCATATTGACAACAGTGCCAGTAGCACCTCCTCCTGCCTCCACCAATCGGCAGTCAGGAAGACAGCCTACTCCCATCTCTCCACCTCTAAGAGACAGTCATCCTCAGGCCATGCAGTGGAACTCCACAACATTCCACCAAGCTCTGCTAGACCCCAGAGTGAGGGGCCTATACTTTCCTGCTGGTGGCTCCAGTTCCGGAACAGTAAACCCTGTTCCGACTATTGCCTCACCCATATCGTCAATCTTCTCGAGGACTGGGGACCCTGCACCGAACATGGAGAACACAACATTAGGATTCCTAGGACCCCTGCTCGTGTTACAGGCGGGGTTTTTCTTGTTGACAAGAATCCTCACAATACCACAGAGTCTAGACTCGTGGTGGACTTCTCTCAATTTTCTAGGGGGAGCACCCACGTGTCCTGGCCAAAATTCGCAGTCCCCAACCTCCAATCACTCACCAACCTCTTGTCCTCCAATTTGTCCTGGCTATCGCTGGATGTGTCTGCGGCGTTTTATCATATTCCTCTTCATCCTGCTGCTATGCCTCATCTTCTTGTTGGTTCTTCTGGACTGCCACGGTATGTTGCCCGTTTGTCCTCTACTTCCAGGAACATCAACTACCAGCAAGGGACCATGCAAGACCTGCACGAATCCTGCTCAAGGAACCTCTATGTTTCCCTCTTGTTGCTGTACAAAACTTTCGGACGGAAACTGCACTTGTATTCCCATCCCATCATCCTGGGCTTTCGCAAAATTCCTATGGGAGTGGGCCTCAGTCCGTTTCTACTGGCTCAATTTACTAGTGCCATTTGTTCAGTGGTTCGTAGGGCTTTCCCCCACTGTTTGGCTTTCAGTTATATGGATGATGTGGTATTGGGGGCCAAGTCTGTACAACATCTTGAGTCCCTTTTTACCTCTATTACCAATTTTCTGTTGTCTTTGGGTATACATTTGAACCCTAATAAAACTAAACGTTGGGGCTACTCCCTTAATTTCATGGGATATGTAATTGGAAGTTGGGGTACCTTACCGCAAGAACACATTGTACTCAAACTCAAGCAATGTTTTAGAAAACTGCCTGTAAATAGACCTATTGATTGGAAAGTATGTCAGAGAATTGTGGGTCTTTTGGGTTTTGCTGCCCCTTTTACACAATGTGGTTATCCTGCCTTAATGCCTTTATATGCATGTATACAATCCAAGCAGGCTTTCACTTTCTCGCCAACTTATAAGGCCTTTCTGTGTAAACAATATCTGCACCTTTACCCCGTTGCCCGGCAACGGTCAGGTCTCTGCCAAGTGTTTGCTGACGCAACCCCCACTGGATGGGGCTTGGCTATTGGCCATCGCCGCATGCGTGGAACCTTTGTGGCTCCTCTGCCGATCCATACTGCGGAACTCTTAGCAGCTTGTTTTGCTCGCAGCCGGTCTGGAGCAAAACTTATCGGAACCGACAACTCTGTTGTCCTCTCTCGGAAATACACCTCCTTTCCATGGCTGCTAGGGTGTGCTGCCAACTGGATCCTGCGCGGGACGTCCTTTGTCTACGTCCCGTCGGCGCTGAATCCCGCGGACGATCCGTCTCGGGGTCGTTTGGGACTCTACCGTCCCCTTCTTCGTCTGCCGTTCCGGCCGACCACGGGGCGCACCTCTCTTTACGCGGTCTCCCCGTCTGTGCCTTCTCATCTGCCGGACCGTGTGCACTTCGCTTCACCTCTGCACGTCGCATGGAGACCACCGTGAACGCTCACCAGGTCTTGCCCAAGGTCTTACATAAGAGGACTCTTGGACTCTCAGCGATGTCAACGACCGACCTTGAGGCATACTTCAAAGACTGTTTGTTTAAGGACTGGGAGGAGTTGGGGGAGGAGATTAGGTTAAAGGTCTTTGTACTAGGAGGCTGTAGGCATAAATTGGTCTGTTCACCAGCACCATGCAACTTTTTGGCCCGAAACCAGAC

**References**

Chen, Y., Qian F Fau - Yuan, Q., Yuan Q Fau - Li, X., Li X Fau - Wu, W., Wu W Fau - Guo, X., Guo X Fau - Li, L., and Li, L. Mutations in hepatitis B virus DNA from patients with coexisting HBsAg and anti-HBs.

Yuan, Q., Ou Sh Fau - Chen, C.-R., Chen Cr Fau - Ge, S.-X., Ge Sx Fau - Pei, B., Pei B Fau - Chen, Q.-R., Chen Qr Fau - Yan, Q., Yan Q Fau - Lin, Y.-C., Lin Yc Fau - Ni, H.-Y., Ni Hy Fau - Huang, C.-H., Huang Ch Fau - Yeo, A.E.T., Yeo Ae Fau - Shih, J.W.K., Shih Jw Fau - Zhang, J., Zhang J Fau - Xia, N.-S., and Xia, N.S. Molecular characteristics of occult hepatitis B virus from blood donors in southeast China.
